# Seroprevalence of measles and natural rubella antibodies among children in Bangui, Central African Republic

**DOI:** 10.1186/1471-2458-11-327

**Published:** 2011-05-17

**Authors:** Alexandre Manirakiza, Jean Marie Kipela, Stephen Sosler, Régis M'Bary Daba, Ionela Gouandjika-Vasilache

**Affiliations:** 1 Institut Pasteur de Bangui, Réseau International des Instituts Pasteur, BP 923, Bangui, Central African Republic; 2 World Health Organization, Libreville, Gabon; 3 World Health Organization, New Delhi, India; 4 World Health Organization, Bangui, Central African Republic

## Abstract

**Background:**

Passively acquired maternal antibodies are necessary to protect infants against circulating measles virus until they reach the eligible age of vaccination. Likewise, high levels of population immunity must be achieved and maintained to reduce measles virus transmission. This study was undertaken to (1) assess the presence of maternally acquired measles-specific IgG antibodies among infants less than 9 months of age in Bangui, Central African Republic and (2) determine the immune status of vaccination-age children and the concordance with reported vaccination status. A secondary objective was to describe the presence of rubella-specific IgG antibody in the study population.

**Methods:**

Vaccination history and blood samples were collected from 395 children using blotting paper. Samples were analyzed for the presence of measles-specific IgG antibodies using commercial ELISA kits.

**Results:**

Measles-specific IgG antibodies were detected in 51.3% of vaccinated children and 27.6% of non-vaccinated children. Maternally derived measles IgG antibodies were present in only 14.8% of infants aged 0-3 months and were absent in all infants aged 4-8 months. The presence of IgG-specific measles antibodies varied among children of vaccination age, from 57.3% for children aged 9 months to 5 years, to 50.6% for children aged 6-9 years and 45.6% for chidren aged 10 years and above. The overall prevalence of rubella-specific IgG was 55.4%, with a high prevalence (87.4%) among children over 10 years of age.

**Conclusion:**

The findings suggest that despite efforts to accelerate measles control by giving a second dose of measles vaccine, a large number of children remain susceptible to measles virus. Further research is required to determine the geographic extent of immunity gaps and the factors that influence immunity to measles virus in the Central African Republic.

## Background

Since 2000, significant progress has been made to reduce the global burden of measles. Nowhere has this achievement been more pronounced than in Africa. Through effective implementation of WHO- and UNICEF-recommended control strategies, the estimated measles-related mortality decreased by 90% in the African Region between 2000 and 2008. In particular, a second dose of measles-containing vaccine (MCV) given during supplemental immunization activities played a vital role in raising population immunity levels, although routine vaccination coverage remains low and health systems weak.

In spite of the gains, measles morbidity and mortality continue to be an important public health concern for Africa's impoverished children, who have poorer access to vaccination and curative services. Moreover, the recent resurgence of measles outbreaks, cases and deaths is a necessary reminder of how short-lived immunity gains and progress can be [1]. All countries in the WHO African Region now offer a second dose of MCV, typically by organizing periodic supplemental immunization activities. Where routine immunization coverage remains low and systems are weak, supplemental activities have been shown to be an effective mechanism for raising population immunity levels.

The Central African Republic (CAR) is a landlocked country in central equatorial Africa that borders five other countries: Chad to the north, Sudan to the east, the Democratic Republic of the Congo and the Congo to the south, and Cameroon to the west. The estimated total population in 2008 was 4.4 million, approximately 20% of whom live in the capital, Bangui. CAR is one of the poorest countries in the region and the world, with a human development index of 0.369, ranking 179 out of 182 countries [2]. The estimated mortality rate of children under 5 was 172 per 1000 live births in 2008 [3]

As in the rest of the African Region, measles control in CAR improved markedly during the past decade. In 2000, there were 3207 reported measles cases, and the measles vaccination coverage was 36% [WHO-UNICEF Best Estimates]. Between 2000 and 2005, routine measles vaccination coverage made steady gains, and it was estimated to be 62% in 2005. In late October 2005, the country initiated second-dose measles catch-up vaccination campaigns as part of the Measles Initiative's efforts to reduce measles-related mortality (CAR Ministry of Health data). The nationwide campaign was conducted in two phases (November 2005 and January-February 2006) and targeted all children aged between 6 months and 14 years. Overall, 1.7 million children were vaccinated, and administrative coverage was estimated to be greater than 90%.

Measles surveillance with laboratory-based serology to confirm outbreaks was launched in 2003. Previously, cases were reported on the basis of a clinical case definition of fever plus rash and either cough, conjunctivitis or coryza. As recommended in the WHO Regional Office for Africa's standard diagnostic protocol, suspected cases that meet the definition of a clinical case of measles and test negative for measles are analyzed differentially for the presence of rubella IgM antibodies. This secondary information provides important epidemiologic data on rubella virus transmission, which previously was unrecognized and poorly understood. The Institut Pasteur de Bangui is the National Measles Reference Laboratory for the Ministry of Health. After the measles catch-up campaigns of 2005-2006, case-based laboratory supported measles surveillance was rolled out in earnest to the entire country.

A more direct and complementary manner of assessing vaccination activities and the level of community protection against measles is to evaluate the immunoglobulin response of a population to a given vaccine antigen. In addition, by including pre-vaccine eligible infants (<9 months of age), this study seeks to improve understanding of the duration of passively acquired maternal protection to measles virus in the CAR context.

Thus, the primary objectives of this study were to assess the presence of maternally acquired measles-specific antibodies in infants not yet eligible for MCV in routine services (children <9 months of age) in the capital city, Bangui and to determine the immune status of vaccination-age children and the association with reported vaccination status. A secondary objective of this investigation was to describe the presence of rubella-specific IgG antibody in the study population.

## Methods

### Study design and population

This study was conducted in Bangui, the capital of CAR, between June and December 2008. The study participants were drawn from a list of 11 pre-determined clinics, hospitals and primary and secondary schools. Healthy children aged 1 month to 15 years were eligible. Children with recently reported acute infectious or chronic disease (i.e. cardiopathy, bronchitis or renal disease), malnutrition, or sickle-cell disease or with HIV infection were excluded from the study. Written informed consent for children's participation in the study was obtained from parents and legal guardians before the start of the study. This study was ethically approved by the local review board of the University of Bangui Medical Research Committee.

A trained investigator used a standardized questionnaire to collect information from parents or legal guardians about age, sex, vaccination status and date of the last dose of vaccine received. Verbal vaccination history was cross-checked with a child's immunization card when available.

A non-probability quota sample was used to sample study participants. The number of participants was determined after assuming a confidence interval of 5% and desired 1-ß (Power) of ≥80% when comparing proportions between age groups. Thus, the minimum sample size for the study was 384 children.

### Sample testing

Blood samples were collected on filter paper from a finger or heel (children <6 months) prick and transported to the Institut Pasteur de Bangui, where they were analyzed for measles and rubella. Measles- and rubella-specific IgG antibodies were detected with enzyme-linked immunosorbent assays (ELISAs), Platelia™ Measles IgG (Bio-Rad, Ref 72686) and Platelia ^® ^Rubella IgG TMB (Bio-Rad, Ref. 72912). The presence of IgG antibodies was determined by comparing the optical density (OD) to a standard range. For measles, a qualitative result was obtained by comparing the patient OD to the cut-off OD (index). Results were interpreted as negative if the index was <0.8, doubtful if it was between 0.8 and 1.2 and positive if it was >1.2, indicating protective antibodies to measles as per the kit instructions.

For rubella, the OD was converted into international units per milliliter by plotting a standard curve for each assay. Results were interpreted as negative (titer ≤10 UI/ml), doubtful (titer 10-15 UI/ml) or positive (titer ≥15 UI/ml) according to the kit instructions. For both measles and rubella, serum samples with doubtful results were automatically retested. If the same result was obtained, the case was classified as negative for the purposes of this study.

### Data management

Data were entered, verified for consistency and analyzed with EpiInfo software version 3.5.1 (CDC, Atlanta, Georgia, USA). Results are expressed as numbers and percentages. Chi-square tests were performed to assess differences between groups. A p value of < 0.05 was considered statistically significant.

## Results

### Study population characteristics and vaccination status

The study population consisted of 395 children recruited from the outpatient care wards of two health centers (42.8%) and seven kindergartens, primary and secondary schools (57.2%). The breakdown of study subjects on the basis of age is shown in Table 1. Slightly more girls than boys (53% vs. 47% or sex-ratio M/F = 0.9) participated in the study. The youngest age group, infants aged 0-8 months, was categorized as unvaccinated, as routine immunization with measles vaccine in the national guidelines is performed only for infants from 9 months of age. The mean and median ages of the study subjects were 64.7 and 55 months, respectively. This did not differ by sex.

**Table 1:** Vaccination status of study subjects during routine immunization and SIA by age

Age group	Vaccination in RI*		Vaccination during SIA**		Possession of vaccination card		Total
	
	n	%	n	%	n	%	
0-8 months	3	2.3%	0	0.0%	133	100.0%	133

9-59 months	61	81.3%	42	56.0%	37	49.3%	75

6 - 9 years	58	69.9%	56	67.5%	14	16.9%	83

≥10 years	33	32.0%	52	50.5%	5	4.9%	103

Total	155	39.3%	150	38.1%	189	48.0%	394

*RI = routine immunization; **SIA = supplementary immunization activities

The reported vaccination coverage with routine services was 39.5% (156/395). When only eligible children were considered (infants aged ≥9 months), the reported routine coverage increased to 58.2% (152/261). Coverage increased further to 83.1% when allowing for either routine or the catch-up campaign (217/261), as per verbal reports from care givers. Nearly all infants under 1 year of age had a routine immunization card (147/148). As expected, possession of an immunization card decreased with increasing age, and only 4.9% (5/103) of the children aged ≥10 years had an immunization card. Details of the vaccination status of study subjects, by age, are presented in Table 1.

### Measles-specific antibody response among infants 0-8 months of age

**Figure 1:**
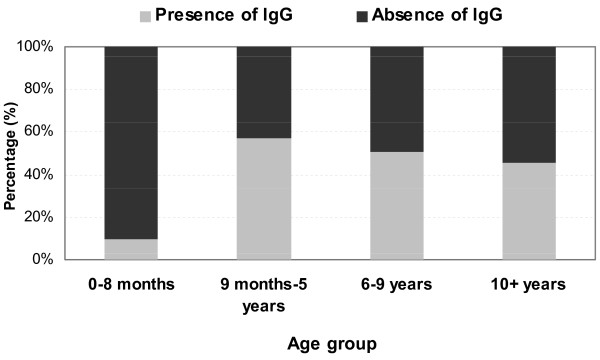
**Presence of measles-specific IgG antibody, infants aged 0-8 months**.

Of the 133 infants aged 0-8 months enrolled in the study, 13 (9.8%) had measles-specific IgG antibody. IgG antibodies were found only in neonates and infants aged 1-2 months. No measles-specific IgG antibody was detected among infants aged 3-8 months. Although three infants in this age group (one aged 6 months and two aged 8 months) had documented proof of measles vaccination, none of these infants had detectable measles-specific IgG antibodies (Figure 1).

### Measles-specific antibody response among children >9 months of age

**Figure 2:**
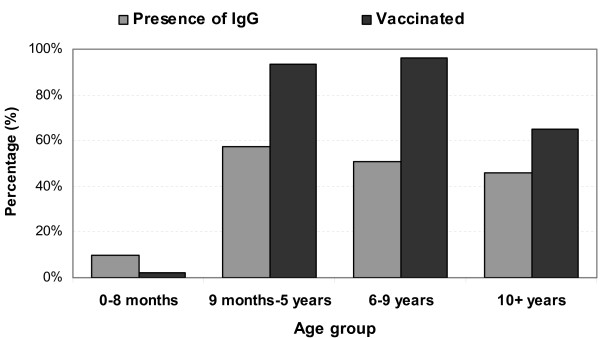
**Presence of measles specific IgG antibody and reported vaccination by age group**.

The presence of measles-specific IgG antibody increased with age to 57.3% (43/75) for children aged 9 months to 5 years. For older children, however, there was a general decrease in IgG presence by age group to 50.6% (42/83) for those aged 6-9 years and 45.6% (47/103) for chilren aged ≥10 years. Among children aged 9 months and older, vaccinated children were more likely to have detectable measles-specific IgG antibodies than unvaccinated children (53.5% vs. 36.4%; chi-square = 4.28; p = 0.04). See Figure 2.

### Rubella-specific antibodies

**Figure 3:**
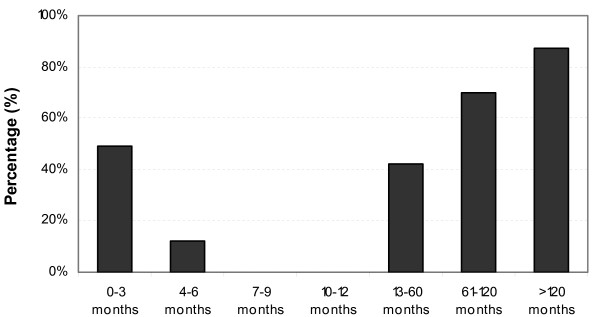
**Natural rubella IgG responses by age group**.

More than the half of all studied children (55.4%; 219/395) had rubella-specific antibodies. Presence of rubella-specific IgG antibodies increased with increasing age of study subjects with protective antibody in 38% of children 1-4 years of age, 71% of children 5-9 years of age, and 87% of children 10 years of age and older. Of 148 children <12 months of age, 31.8% had rubella-specific IgG antibodies but none of the children 7-11 months of age (0/23) had detectable antibody. Presence of rubella-specific IgG antibodies was distributed equally between males and females (male: 44.1%, female: 55.9%; p = 0.9). See Figure 3.

## Discussion

This study documents for the first time the seroprevalence of measles-specific IgG antibodies among children in the CAR. The results suggest that population immunity to measles vaccine is well below the threshold (>90%) necessary to interrupt measles transmission. Protective antibody levels were more prevalent among children eligible for vaccination and among those reported to have been vaccinated; however, only 50% of vaccination-age children had protective antibodies. Passively derived maternal protection to measles vaccine among infants aged 0-8 months was also low (9.8%) and undetectable past 3 months of age, leaving this vulnerable population susceptible to wild virus circulation. The reasons for low maternal transfer of measles-specific antibody and the lower-than-expected population protection are not clear.

Low, waning maternal antibody protection, which results in an increased period of susceptibility to natural infection before vaccination eligibility, typically at 9 months of age, has been documented in various developing countries [4-9]. In a recent study in Bangladesh, only 50% of infants aged 1-3 months had protective antibodies and infants aged >3-9 months had no protection at all, similar to our findings [8]. Two studies conducted in the past 10 years in sub-Saharan Africa reported similar results. In Nigeria, Hartter et al. found that only 17% of 4-month-old Nigerian infants were still protected against measles [7], while in Mali, only 30% of 2-month-old infants had protective antibody titers [9]. Reporting results from Libreville, Gabon, more than 20 years ago, Grendel et al. found that 90% of children consulting outpatient services were seronegative by 4 months of age [5]. During the same period, Dabis et al. found rapid rates of passive maternal antibody loss, from 96% at 2 months of age to less than 10% at 7-8 months of age in Brazzaville, Congo [4].

This body of research presents the accumulated descriptive epidemiological evidence on this topic. Unfortunately, there remains a dearth of analytic research in Africa to determine the role and relative importance of specific covariates associated with this phenomenon. Furthermore, the epidemiologic context of measles transmission has changed dramatically in the past decade [10]. Accelerated measles control activities, including improved routine immunization coverage, the provision of a second dose of measles vaccine at supplementary vaccination activities and case-based surveillance with laboratory confirmation, have reduced measles-associated morbidity and mortality to record lows. Bolstered by these achievements, the African Region is poised to adopt an elimination goal. Our results are important, because they provide a rare glimpse into the serological profile of measles protection in Africa after concerted, targeted efforts to raise population immunity and reduce morbidity and mortality.

Factors known to affect the placental transfer of antibodies, such as HIV and malaria infection, may be associated with our study results [11]. CAR has an estimated HIV seroprevalence rate of 6.2%, one the highest in central Africa [12]. Attempts were made to ascertain the HIV status of potential study subjects; however, serological screening was not undertaken. Falciparum malaria transmission is haloendemic in much of the country, including the capital Bangui, the site of the study. Maternal histories of malaria episodes were not collected.

UNICEF estimates that 38% of the country's children suffer from global chronic malnutrition and 10% suffer from global acute malnutrition [13]. While the effect of malnutrition on maternal transfer of passive antibody immunity is poorly understood, the scope and severity of the problem indicate that it cannot be ignored. The source of maternal immunity, wild virus versus vaccine-derived, may also affect the amount of maternal antibody transferred transplacentally, with vaccinated mothers passing on less immunity to their newborn infants. Although routine vaccination rates increased from 35% in 2003 to 62% in 2006, before this, routine vaccination with measles antigen remained consistently below 50%. For this reason, it is believed that the majority of maternal immunity is likely derived from exposure to circulating wild virus.

Our study also suggests lower-than-expected levels of measles-specific IgG in the vaccination-eligible population, and even a possible waning of serological immunity among children aged ≥10 years. This is another result that deserves further investigation. Reduced vaccine effectiveness, due to compromised vaccine or host characteristics that negatively affect seroconversion as well as reduced exposure to circulating measles virus may all play a role.

This study has some potential limitations. The presence of protective antibodies may have been misclassified because of the diagnostic test used. The selection of study subjects may also have biased our results. Although children with recent episodes of acute illness were ineligible for the study, some were nonetheless recruited from outpatient clinic services. In addition, the quota convenience sampling method used does not allow results to be extrapolated to the wider population. Determining the vaccination status of children and the reliability of reports is always problematic in the absence of the gold standard vaccination card. Thus, the vaccination status of study subjects may have been overestimated. Lack of verification of the vaccination status of the mothers is another weakness of this study.

National immunization programs must determine the optimal age for the first dose of MCV1 by weighing the risk for primary vaccine failure at younger ages against the risk for measles virus infection before vaccination. This study describes the wide window of vulnerability, between the disappearance of passively acquired maternal antibody and the eligible age of vaccination. The premature loss of maternal antibodies, which, if present, would inhibit vaccination seroconversion, opens the door to discussions of the optimal age for vaccination in developing countries.

## Conclusion

The prevalence of measles IgG-specific antibodies of study children in Bangui is low, indicating that a large number of children may not be protected against measles. The rapid loss of maternal protective antibodies opens a wide period of vulnerability of infants and suggests that the first dose of measles vaccine could be administered at an earlier age. Vaccine effectiveness studies in younger age groups with an additional dose administered at or close to 12 months, when vaccine effectiveness is higher, could be tested. In the meantime, the quality of vaccination activities (routine and supplementary immunization campaigns) must be improved, and population awareness of their importance must be increased. Monitoring routine immunization coverage to assess administrative coverage levels, which are habitually unreliable, and robust methods to monitor and evaluate supplementary immunization activities are needed. Complementary sero-immunity studies to detail the dynamics of measles and rubella IgG antibody responses in other areas of the CAR should be conducted to better understand the window of vulnerability of infants and the immunity profile of old age groups throughout the country. In addition, rubella control and congential rubella syndrome prevention (CRS) through the use of rubella containing vaccine should be considered as per WHO regional and global guidelines. Finally, as the routine immunization system continues to improve and higher coverage is achieved, the introduction of a second dose of measles containing vaccine into the routine immunization schedule should be considered.

## Competing interests

The authors declare that they have no competing interests.

## Authors' contributions

IGV and JMK conceived the study, with substantial contributions from AM, SS and RMD. Field data were collected under the supervision of IGV. AM, SS, JMK, and IGV analyzed and interpreted the data and wrote the manuscript. All authors read and approved the final version of the manuscript.

## Pre-publication history

The pre-publication history for this paper can be accessed here:

http://www.biomedcentral.com/1471-2458/11/327/prepub
